# Diagnostic delay in patients diagnosed with Lyme carditis presenting with cardiac symptoms

**DOI:** 10.1016/j.jccase.2025.03.011

**Published:** 2025-04-17

**Authors:** Jon Are Sørås, Gard Frodahl Tveitevåg Svingen, Terje H. Larsen, Håvard Keilegavlen, Trygve Kristiansen, Øystein Wendelbo

**Affiliations:** a Department of Medicine, Haukeland University Hospital, Bergen, Norway; b Department of Heart Disease, Haukeland University Hospital, Bergen, Norway; c Department of Biomedicine, University of Bergen, Bergen, Norway; d VID Specialized University, Faculty of Health, Bergen, Norway

**Keywords:** Lyme disease, Lyme carditis, Arrhythmia, Diagnostic delay

## Abstract

In this case report, we describe two previously healthy young males who presented with cardiac symptoms suggestive of Lyme carditis (LC). LC-associated arrhythmia is a potentially fatal complication of Lyme disease, which typically occurs during the early disseminated and late stages. In high endemic areas a high degree of suspicion is vital to avoid misdiagnosis and delayed treatment, and to prevent long-term complications of disseminated infection and potentially fatal outcome.

**Learning objective:**

Lyme carditis (LC) can present with a wide array of symptoms. The following two cases illustrate the diverse clinical manifestations of LC, as well as the potential for ‘doctor's delay’ in diagnosing patients with LC.

## Introduction

*Borrelia burgdorferi* sensu lato is a collective term for several *Borrelia* species, the causative agent of Lyme disease (LD), and is transmitted to vertebrate hosts by *Ixodes* ticks. Untreated, LD can cause disease manifestations in several organs, including the heart [1].

Lyme carditis (LC) is a potentially fatal complication of LD. In Europe, LC is a rare manifestation of untreated LD, with regional differences and a reported incidence of 0.3–4.0 % [2]. The most common clinical presentation is high-degree atrioventricular (AV) block, which can progress rapidly [3]. Most AV blocks resolve with appropriate antibiotic treatment without the requirement for a permanent pacemaker [4].

This case report discusses two young previously healthy males presenting with cardiac symptoms and AV block. However, neither patient could recall previous tick bites or the pathognomonic erythema migrans (EM), characteristic for the early localized stage of LD. Thus, correct diagnosis and appropriate treatment were delayed in both patients.

## Case report

### Patient A

An 18-year-old male, residing in western Norway, was admitted to the emergency department (ED) of our regional University Hospital in July 2020 after three weeks of periodic heart palpitations, dyspnea, and dizziness. He had no previous medical history, recent travel history, and did not take any regular medications.

**Fig. 1:**
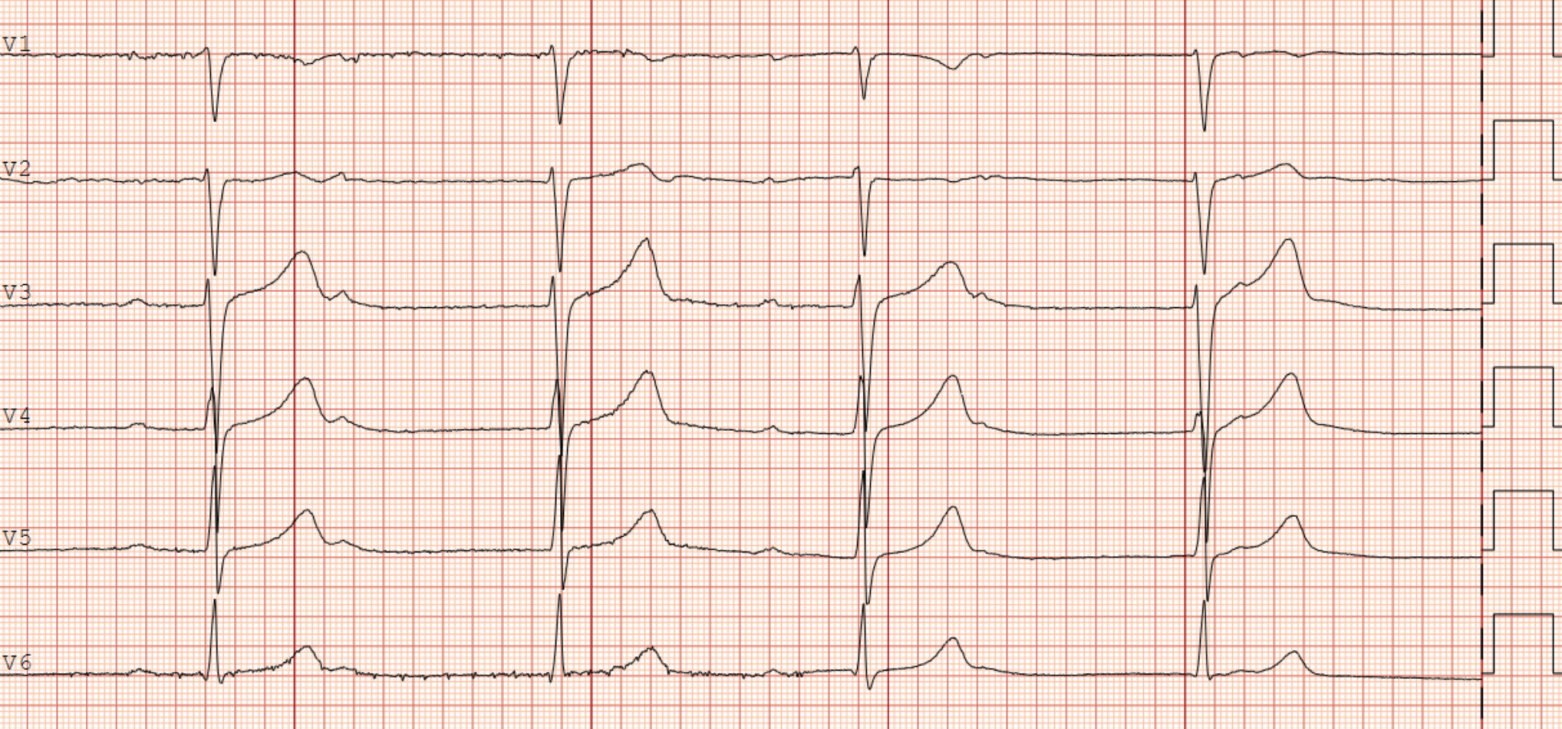
Second-degree AV block, Mobitz type 1. 12‑lead ECG of an 18-year-old patient on admission to the emergency department of our hospital. Shown here are the precordial leads, revealing sinus rhythm with second degree AV block, Mobitz type I. The QRS complexes are narrow. The ECG speed is 50 mm per second. AV, atrioventricular; ECG, electrocardiogram.

On admission to the ED, the patient reported no symptoms but recalled an unspecific insect bite on his ankle one month prior to admission, with subsequent development of an itchy, red rash around the bite, lasting for two weeks. Clinical examination and vital parameters were unremarkable. Blood tests showed a slightly elevated N-terminal pro-B-type natriuretic peptide (NT-proBNP) level of 709 ng/L (reference range < 85 ng/L). The electrocardiogram (ECG) (Fig. 1) showed second-degree AV block Mobitz type I. Transthoracic echocardiography (TTE) demonstrated slightly reduced left ventricular ejection fraction of 45–50 %, but was otherwise normal. The patient was transferred to the cardiovascular intensive care unit for closer monitoring.

Within hours, the patient reported dizziness while lying flat on his back, and the cardiac telemetry revealed third-degree AV block with a junctional escape rhythm of 26 beats per minute. To maintain an adequate cardiac output, a continuous intravenous isoprenaline infusion was initiated, stabilizing the junctional escape rhythm at 60 beats per minute and improving the patient's symptoms.

The patient's progressive AV block, previous reported insect bite, and the fact that the patient resided in an LD endemic region, led to the suspicion of LC as the underlying cause. A *Borrelia burgdorferi* antibody test was performed and came out positive (IgM 145 AU/mL, IgG 1297 AU/mL) on day two after admission. The patient was started on ceftriaxone 2 g intravenously every 24 h.

Due to hemodynamic instability and continuous need of isoprenaline, a temporary transvenous pacemaker (PM) was placed on day 3. The AV block gradually resolved during the following week, and on day 10 of admission the temporary transvenous PM was removed. The patient was discharged the following day with a one-week course of 100 mg oral doxycycline twice daily. His general practitioner followed the patient after discharge from the hospital, and the patient has had no further reported cardiovascular admissions or events in the three years since discharge.

### Patient B

A 19-year-old male was admitted to his local hospital in western Norway in July 2020 with a one-day history of inspiratory chest pain along with intermittent pressure in the chest radiating to his right upper arm. He had no previous medical history and did not take any regular medications or illicit drugs.

On admission, the patient had a temperature of 38.7 °C. Remaining vital parameters and the clinical examination were normal. Blood tests revealed an elevated leukocyte count of 12.3 /L (reference range 4.1–12.0 × 10^9^ /L) along with a high-sensitivity cardiac troponin T (hs-cTnT) of 190 ng/L (reference range < 15 ng/L). The ECG showed sinus rhythm with normal AV conduction. Chest X-ray and TTE were described as normal. A non-ST-elevation acute coronary syndrome (NSTE-ACS) was suspected, and the patient was transferred to our hospital for urgent coronary angiography.

Ten hours after initial presentation, the patient was admitted to our hospital. The ECG now showed sinus rhythm with ST elevations in leads I, aVL, and V4 to V6, along with a mild PR-segment depression. Hs-cTnT was 195 ng/L.

**Fig. 2:**
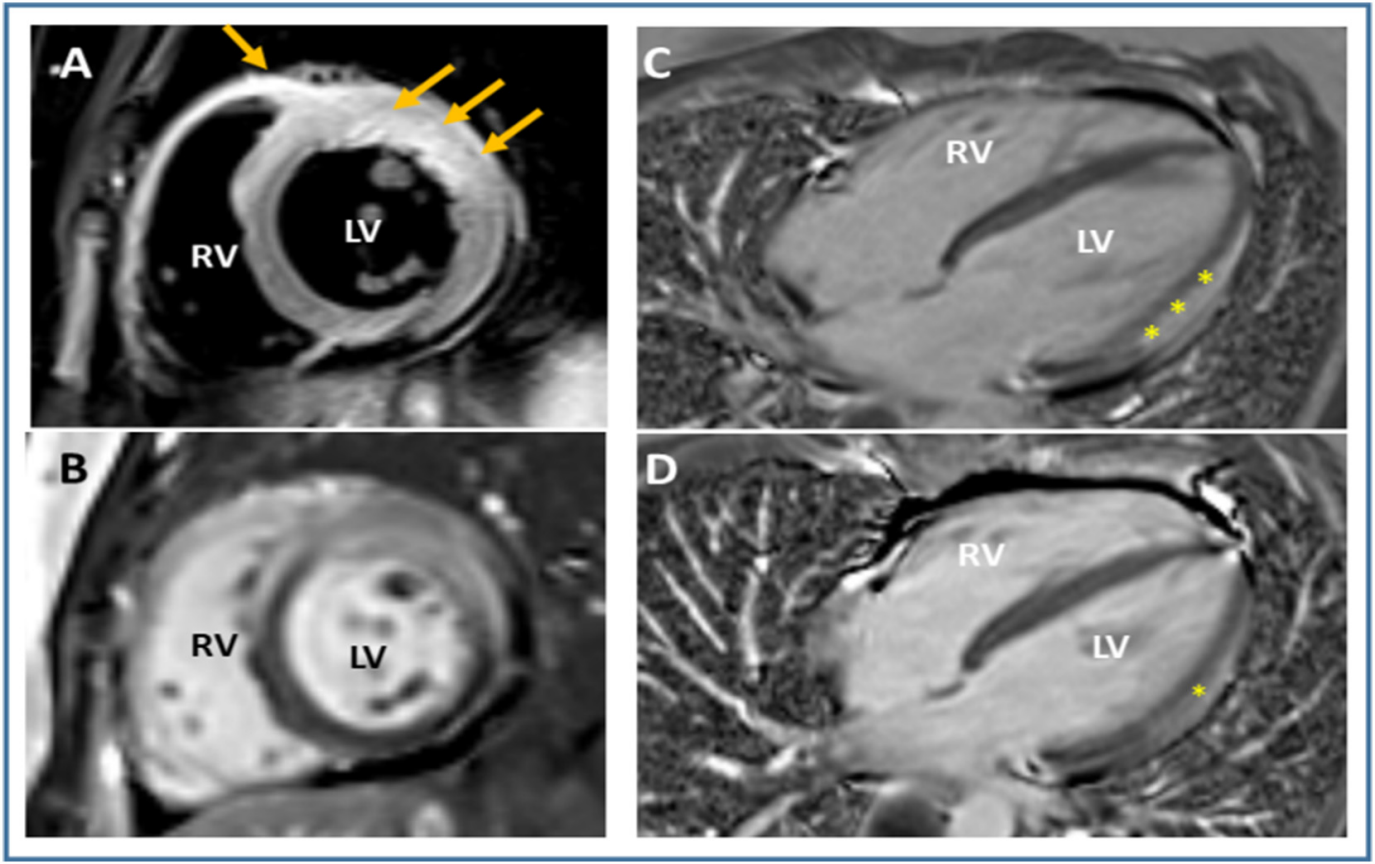
Initial CMR (A-C) and follow-up CMR at 3 months (D). Short-axis T2-weighted short-tau inversion recovery view (A) shows severe edema (arrows) in the anteroseptal, anterior, and anterolateral wall of the left ventricle (LV) as well in the anterior free wall of the right ventricle (RV). In addition, the pericardium covering the affected myocardium shows increased signal and thickening. Distribution of LGE (B) corresponds well with extent of edema, and these changes appear mainly transmurally across the anterior wall. In the 4-chamber-view, LGE (asterisks) is initially (C) extensively distributed in the midmyocardial and subepicardial layers of the LV wall and in the pericardium covering the lesion. At follow-up CMR (D) the presence of LGE is considerably reduced. CMR, cardiac magnetic resonance imaging; LGE, late gadolinium enhancement.

Coronary computed tomography angiography (CTA) showed no coronary stenoses. A cardiac magnetic resonance imaging scan (CMR) showed extensive myocardial edema and late gadolinium enhancement (LGE) in the left ventricle (LV) as well as in the free wall of the right ventricle (RV). In addition, signs of inflammation were seen in the pericardium covering the affected myocardium. No significant pericardial effusion was detected (Fig. 2A-C). The left ventricular ejection fraction as measured by the CMR was 58 %. A non-specific viral perimyocarditis was suspected. Treatment with ibuprofen 600 mg three times daily and colchicine 500 μg twice daily was prescribed, and the patient was discharged from hospital after seven days.

The patient was readmitted two weeks after discharge, with a one-week history of chest pain and heart palpitations. An ECG showed alternating first-degree AV block and Mobitz type I second-degree AV block. The patient was admitted to the cardiology ward, where telemetry monitoring revealed episodes of Mobitz type II second-degree AV block. The next morning the monitor revealed episodes of advanced AV block with pauses lasting up to 4 s. The patient reported nausea and palpitations that correlated to his bradycardia. Due to progressive AV block, a temporary transvenous PM was implanted.

The patient resided in an LD endemic region but could not recall ever experiencing a tick bite, but due to the patient's symptoms serologic testing for *Borrelia bugdorferi* was performed and came back positive (IgM 1160 AU/mL, IgG 1205 AU/mL). Intravenous ceftriaxone was initiated but changed to doxycycline 100 mg twice daily following a suspected drug reaction. The patient gradually improved over the next five days and regained sinus rhythm. The temporary PM was removed on day 5, and the patient was discharged from hospital on day 6.

CMR follow-up after 3 months revealed complete normalization of myocardial edema and regional hypokinesia whereas some LGE still persisted (Fig. 2D). Follow up ECG and TTE one year after the first admission were described as normal.

## Discussion

The number of LD cases in Europe has increased steadily, with >360,000 cases reported over the past 20 years [5], and an incidence of >10 per 100,000 population per year (PPY). In Norway, the overall incidence is <10 per 100,000 PPY, with higher rates along the southwestern coastline [6].

We describe two previously healthy young males, both admitted to Norwegian ED in July 2020 with LC as a part of an early-disseminated stage of LD. Neither of the patients could recall previous tick bites or the pathognomonic EM, although patient A had experienced a recent, unspecified insect bite.

For several reasons a final diagnosis of LC was delayed in both cases. For patient A delayed treatment occurred awaiting confirmation of *Borrelia* serology. For patient B, a diagnosis of NSTE-ACS was initially suspected, followed by a suspected viral perimyocarditis once atherosclerotic coronary disease was ruled out. On patient B's second admission, targeted antibiotic treatment was delayed while awaiting antibody testing.

According to Scheffold et al. [4], the diagnostic triad of LC comprises medical history (i.e. EM, tick bite), AV block, and a positive Borrelia serology. Both patients had high levels of *Borrelia bugdorferi* specific antibodies in serum detected with Liaison® chemiluminescence immunoassay (CLIA, DiaSorin; Saluggia, Italy), clearly indicative of recent infection. In our patients, there was no evident tick bite. Hence, the usage of the Suspicious Index in Lyme Carditis (SILC) system may have supported the clinicians in evaluating the probability of LC being the cause of the patients' symptoms [7].

LC is often misdiagnosed due to its rarity and nonspecific symptoms [8], requiring high suspicion to avoid delays in diagnosis and treatment. Several diseases can present with both cardiac and skin manifestations (Table 1). While AV block is common in LC, a study of 2899 adults with complete AV block found that only 1.8 % were due to LC [9]. Mortality rates in LC are low [4], albeit rapid progression from constitutional symptoms and AV block to death has been reported [10]. Early recognition and prompt antibiotic therapy are crucial even before final diagnosis has been made.

**Table 1:** Differential diagnosis for patients presenting with both cardiac and skin manifestations.

Disease	Skin manifestations	Cardiac manifestations
Lyme disease	Erythema migrans, borrelial lymphocytoma	Carditis, arrhythmias
Systemic lupus erythematosus (SLE)	Butterfly rash, discoid lesions, photosensitivity	Pericarditis, myocarditis, valvular heart disease
Rheumatic fever	Erythema marginatum	Endocarditis, myocarditis, pericarditis
Sarcoidosis	Lupus pernio, erythema nodosum	Arrhythmias, heart block, cardiomyopathy
Vasculitis (e.g. GPA, EGPA)	Palpable purpura, skin ulcerations	Myocarditis, pericarditis
Infective endocarditis	Janeway lesions, Osler's nodes	Valve dysfunction, heart murmurs
Kawasaki disease	Rash, strawberry tongue, swollen hands/ft	Coronary artery aneurysms, myocarditis
Hypereosinophilic syndrome	Urticaria, angioedema, pruritic papules	Endomyocardial fibrosis, thrombi
Scleroderma (systemic sclerosis)	Skin thickening, Raynaud's phenomenon	Pulmonary hypertension, myocardial fibrosis
Drug-induced lupus	Rash, photosensitivity	Pericarditis, pleuritis

The current case report highlights that patients residing in high endemic regions of LD, presenting with cardiac manifestations such as conduction disturbances with or without myocarditis, should raise an early suspicion of LC. Patients with suspected LC should be treated with appropriate antibiotics not awaiting serologic results.

## Consent

Both patients gave written consent before the writing and publication of this case report.

## Funding statement

No funding.

## Declaration of competing interest

No conflict of interest.
